# MarkovFit: Structure Fitting for Protein Complexes in Electron Microscopy Maps Using Markov Random Field

**DOI:** 10.3389/fmolb.2022.935411

**Published:** 2022-07-25

**Authors:** Eman Alnabati, Juan Esquivel-Rodriguez, Genki Terashi, Daisuke Kihara

**Affiliations:** ^1^ Department of Computer Science, Purdue University, West Lafayette, IN, United States; ^2^ Computer Engineering School, Costa Rica Institute of Technology, Cartago, Costa Rica; ^3^ Department of Biological Sciences, Purdue University, West Lafayette, IN, United States

**Keywords:** protein modeling, cryo-EM, Markov random field, structure fitting, protein structure prediction

## Abstract

An increasing number of protein complex structures are determined by cryo-electron microscopy (cryo-EM). When individual protein structures have been determined and are available, an important task in structure modeling is to fit the individual structures into the density map. Here, we designed a method that fits the atomic structures of proteins in cryo-EM maps of medium to low resolutions using Markov random fields, which allows probabilistic evaluation of fitted models. The accuracy of our method, MarkovFit, performed better than existing methods on datasets of 31 simulated cryo-EM maps of resolution 10 Å , nine experimentally determined cryo-EM maps of resolution less than 4 Å , and 28 experimentally determined cryo-EM maps of resolution 6 to 20 Å .

## 1 Introduction

Proteins are vital components of living cells. Among experimental technologies that are used to determine protein structures, cryogenic-electron microscopy (cryo-EM) has been used in an increasing number of cases as it has notable advantages, including its suitability for determining large macromolecular structures (Kuhlbrandt, 2014; Cheng, 2018; Renaud et al., 2018). The use of Cryo-EM was boosted by the advanced technology of improvements in direct detectors and image processing algorithms (Bai et al., 2015; Nogales, 2016; Wu and Lander, 2020).

To interpret determined cryo-EM maps, computational methods for modeling macromolecule structures play a crucial role (Esquivel-Rodriguez and Kihara, 2013; Alnabati and Kihara, 2019; Malhotra et al., 2019). If a map is determined at a relatively high resolution of up to about 3–4 Å, protein structures can be directly modeled from the density (Terashi and Kihara, 2018; Terwilliger et al., 2018; Terashi et al., 2020). For maps of medium to low resolution (∼4–10 Å and even lower resolution), the density usually does not have sufficient information for *de novo* full-atom modeling. However, protein secondary structures can be captured (Maddhuri Venkata Subramaniya et al., 2019; Wang et al., 2021) and known structures of individual proteins can be fitted to the density maps (Han et al., 2021).

Structure fitting methods have a long history of over two decades, partly because they have been the only option for structure interpretation in the early days when high-resolution maps were not obtained. Methods developed include EMfit (Rossmann et al., 2001), ADP_EM (Garzon et al., 2007), colores (Chacon and Wriggers, 2002), HADDOCK (van Zundert et al., 2015), gmfit (Kawabata, 2008), EMLZerD (Esquivel-Rodriguez and Kihara, 2012), and BCL EM-Fit (Woetzel et al., 2011). As the structure of proteins in the map can be slightly different from those available in an isolated condition, some methods focus more on considering the flexibility of proteins (Tama et al., 2004; Velazquez-Muriel et al., 2006; Velazquez-Muriel and Carazo, 2007; Trabuco et al., 2008; Wang and Schroder, 2012; Lopez-Blanco and Chacon, 2013). To assess the goodness of fit of a structure in a density map, conventionally, the cross-correlation coefficient (CCC) has been used. Several other metrics were also developed, such as core-weighted CCC (Wu et al., 2003), Laplacian-filtered CCC (Vasishtan and Topf, 2011; Joseph et al., 2017), mutual information (Joseph et al., 2017), overlap, and evolutionary information of interface residues (Joseph et al., 2016), and match of density gradient (Han et al., 2021).

Among all the structure fitting methods developed, there are only a few methods developed for multiple chain fitting. γ -TEMPy (Pandurangan et al., 2015) identifies the position of subunits in a cryo-EM map using a vector quantization algorithm, then uses a genetic algorithm to generate different docking conformations and evaluates them using a mutual information score and an atom clash penalty score. Gmfit (Kawabata, 2008; 2018) uses Gaussian mixture models (GMM) to represent subunits and a cryo-EM map. A method developed by Bonomi et al. uses GMMs to represent a cryo-EM map and atomic subunits (Bonomi et al., 2019). Different conformations of subunit interactions are sampled with Replica Exchange Monte Carlo and scored with a Bayesian weighing score which encodes the map density and prior knowledge of domain connectivity. EMLZerD (Esquivel-Rodriguez and Kihara, 2012) constructs a pool of multi-subunit docking models using Multi-LZerD (Esquivel-Rodriguez et al., 2012) and selects ones that have a consistent overall surface shape with the density map using 3D Zernike Descriptors (Kihara et al., 2011). HADDOCK-EM (van Zundert et al., 2015) is another docking-based method that uses additional sources of information such as mutagenesis and cross-link data for docking.

Here, we developed a new method, named MarkovFit, which performs simultaneous-rigid fitting of atomic protein subunits into medium- to low-resolution cryo-EM maps. The method starts by using FFT to search the conformational space for potential positions of subunits and computes scores that quantify the goodness-of-fit between each subunit and the cryo-EM map and the interactions between the subunits. Subsequently, subunits and their physical interactions are represented using a Markov random field (MRF) graph (Besag, 1971). MRF nodes exchange information using a belief propagation algorithm, and the best conformations are extracted using a max-heap tree. Lastly, the top final conformations undergo structural refinement. Taking advantage of MRF, MarkovFit evaluates the fit of individual subunits to the map and subunit interactions efficiently in an integrated fashion. We first benchmarked MarkovFit in comparison with γ -TEMPy on the dataset they used, which includes 10 simulated EM maps. The average root-mean-square distance (RMSD) of MarkovFit was 2.58 Å for the best model among the top 10-scored models and 3.27 Å for the top-scored model, while those of γ -TEMPy were 9.22 Å and 15.32 Å for the best model among the top 10-scored and the top-scored models, respectively. We further compared with Bonomi’s method on their simulated dataset of 19 protein complexes, where MarkovFit had an average RMSD of 2.74 Å and 3.55 Å for the best models among the top 10-scored and top-scored models, respectively, while Bonomi’s method had average RMSDs of 4.86 Å and 5.27 Å for the best models among the top 10-scored and top-scored models, respectively.

We benchmarked MarkovFit on a dataset of nine high-resolution experimentally determined maps in which each subunit was shifted and rotated randomly. The average RMSD was 1.85 Å for the best model among the top 10, which was the same for the top-scored models. We further benchmarked MarkovFit on a dataset consisting of 28 experimental maps of medium to low resolution. The experimental dataset has two versions. In the first version, each subunit was shifted and rotated randomly, while the initial orientation was used in the second version. For the randomly transformed experimental dataset, the average RMSD values were 8.14 Å and 13.91 Å for the best model among the top 10 and top-scored models, respectively. For the non-transformed experimental dataset, the average RMSD values were 6.08 Å and 9.95 Å for the best model among the top 10 and top-scored models, respectively. The source code of MarkovFit was made freely available at https://github.com/kiharalab/MarkovFit.

## 2 Materials and Methods

### 2.1 Overview of MarkovFit Algorithm

**FIGURE 1:**
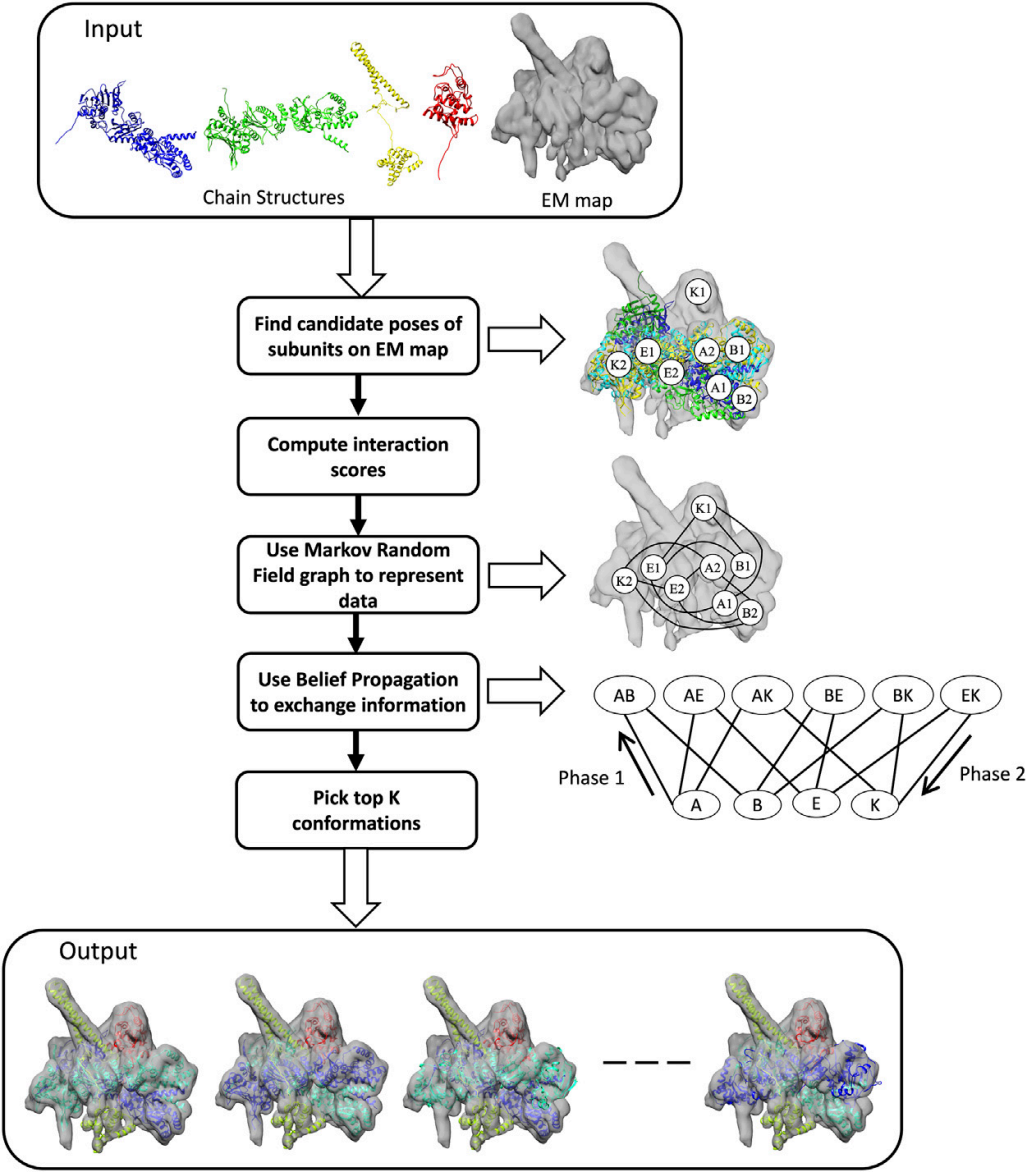
Workflow of MarkovFit. The method starts by searching the conformational space for potential positions of each subunit and computes scores which quantify the goodness-of-fit between each subunit and the cryo-EM map, and the interactions between the subunits. The right panel shows some candidate poses of subunits A, B, E, and K, which are A1, A2, B1, B2, E1, E2, K1, and K2. Subsequently, subunits and their interactions are represented using a Markov random field (MRF) graph. MRF nodes exchange information using a belief propagation algorithm. The graph at the bottom of the right panel shows single nodes, each representing the candidate poses of a subunit, and interaction/pairwise nodes, each representing the interactions between candidate poses of a pair of subunits. The last step is extracting the top conformations using a max-heap tree.

The purpose of MarkovFit is to fit the atomic structures of subunits of a protein complex to a cryo-EM density map so that subunits align well with the map and subunits establish proper physical interactions. As illustrated in Figure 1, the method performs 6D exhaustive searching of the translational and orientational sampling space in the cryo-EM map to compute the candidate positions of each subunit. To accelerate the sampling of the translational space, we used fast Fourier transform (FFT) with a shifting interval of one voxel of the map (it is usually less than 2 Å). To sample the orientational space efficiently, we used a set of quaternions which covers the whole orientation space by 7,416 orientations (Karney, 2007). For each rotation, the search process shifts the rotated subunit structure with the interval and calculates a score at each position, quantifying the goodness of fit of a subunit into the cryo-EM map, which is the sum of cross-correlation and overlap.

The cross-correlation coefficient (CCC) is computed as follows for experimental maps:

$$CCC=\frac{\sum X_{i}Y_{i}}{\sqrt{\sum X_{i}^{2}\sqrt{\sum Y_{i}^{2}}}},$$ (1)

where $X_{i}$ and $Y_{i}$ correspond to the density values of voxel *i* in the experimental map *X* of a protein complex and the simulated map *Y* of the subunit, respectively. For simulated maps, we used the Pearson’s cross-correlation coefficient (PCCC):

$$PCCC=\;\frac{\sum \left(X_{i}-\bar{X}\right)\left(Y_{i}-\bar{Y}\right)}{\sqrt{\sum {\left(X_{i}-\bar{X}\right)}^{2}}\sqrt{\sum {\left(Y_{i}-\bar{Y}\right)}^{2}}}.$$ (2)

Here, $\bar{X}$ and $\bar{Y}$ are the average density values of the two maps. In the case of simulated maps, using the normalized density with PCCC turned out to be more suitable because it can consider regions’ surfaces more effectively without focusing on dense cores in EM maps.

The overlap (OV) score measures the fraction of overlapping voxels between two maps, *X* and *Y*:

$$OV=\;\frac{X\cap \;Y}{Y}.$$ (3)

Any pose that has an overlap <50% with the EM map was excluded. The 50% overlap was chosen arbitrarily as we thought a pose less than 50% overlap would be safe to remove from the consideration. A careful optimization of this cutoff may be able to further improve the accuracy or efficiency of the algorithm. The shifted position of the highest score is selected per rotation. Resulted poses are clustered to remove positions within a predetermined distance (8 Å) from each other, and then they are sorted by the combined score. Then, up to the top 100 poses are selected for each subunit.

Next, for each pair of subunits, interactions are evaluated by a docking score that combines scoring terms, which are a Van der Waals potential, electrostatic interaction, hydrogen and disulfide bonds, solvation, and atom clashes, which we have used for evaluating protein-protein docking (Esquivel-Rodriguez et al., 2012).

Exploring all possible combinations of the subunits is a time-consuming process. To explore the search space efficiently, we use pairwise Markov random field (MRF) in MarkovFit. An MRF is a non-directional probabilistic graphical model where nodes represent poses of subunits in the EM map and edges represent interactions between subunits. A complex conformation is evaluated by the sum of node potentials over single random variables, representing the different conformations of each subunit, and edge potentials over pairs of variables:

$$P\left(X_{1},\ldots ,X_{N}\right)=\;\frac{1}{Z}\prod_{i\in N}\Phi \left(X_{i}\right)\prod_{i,j\in E}\Psi \left(X_{i},X_{j}\right),$$ (4)

where *N* is the number of subunits, *X*_*i*_ is a pose of subunit *i*, *Φ* is the node potential, which considers the goodness-of-fit of each subunit in the pose, *Ψ* is an edge potential, which considers the interaction of subunits, *E* represents all combinations of the subunits, and *Z* is the partition function used for normalization.

To avoid numerical issues that might arise from multiplications, we adopted a log-linear model for the scoring function:

$$\arg max_{X}\propto exp\left[-\sum_{i=1}^{N}w_{cc}f_{cc}\left(X_{i}\right)-\sum_{i=1}^{N}w_{ov}f_{ov}\left(X_{i}\right)-\sum_{\left(i,j\right)\in E}w_{ph}f_{ph}\left(X_{i},X_{j}\right)-\sum_{\left(i,j\right)\in E}w_{cl}f_{cl}\left(X_{i},X_{j}\right)\right],$$ (5)

where CC is cross-correlation, OV is overlap, Ph is the physics-based docking score, CL is the clash score, and *w* is the weight assigned for each score.

The four weights were briefly optimized on five experimental maps, EMDB-ID 0366, 1970, 3340, 2526, and 8475, as follows: First, we tried an equal weight, 1.0 for all the four weights, *f*_*cc*_, *f*_*ov*_, *f*_*ph*_, and *f*_*cl*_. Then, we compared the RMSD of the models with a weight combination of 0.5, 1.0, 1.0, and 1.0, for the four weights, respectively. Furthermore, we used *f*_*cc*_ = 0.5, with exhaustive combinations of 0.8, 0.9, and 1.0 for *f*_*ov*_, *f*_*ph*_, and *f*_*cl*_, respectively; and finally chose to use *f*_*cc*_ = 0.5, *f*_*ov*_ = 0.9, *f*_*ph*_ = 1.0, and *f*_*cl*_ = 0.8 throughout the study. We did not perform extensive optimization because the results were the same for most of the combinations on these five maps.

**FIGURE 2:**
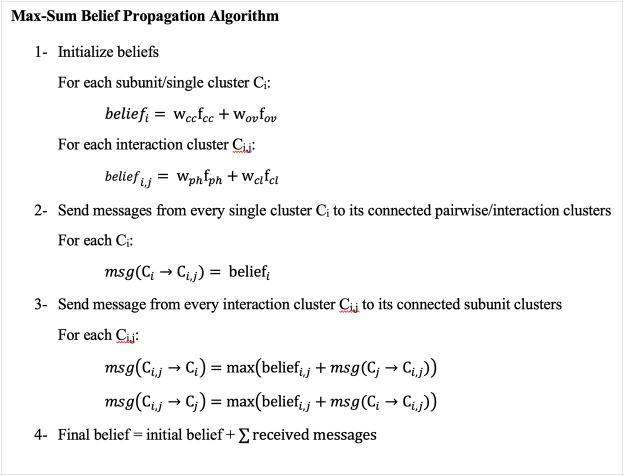
Overview of the Max-Sum Belief Propagation Algorithm. The algorithm has four main steps. 1, it initializes the beliefs of subunit/single clusters (nodes) to be the sum of the weighted goodness-of-fit scores of their candidate poses, and the beliefs of interaction/pairwise clusters (nodes) to be the sum of the weighted pairwise scores between the poses of every pair of subunits. 2, subunit clusters (nodes) send their initial beliefs to their connected pairwise clusters (nodes). 3, pairwise clusters (nodes) send messages to their connected subunit clusters (nodes) containing the sum of the pairwise initial beliefs and the subunit messages they received from subunit clusters (nodes), excluding the receiving cluster (node) messages. 4, subunit clusters (nodes) add pairwise beliefs to their initial beliefs. This algorithm corresponds to the right-bottom graph in Figure 1, which is called a cluster graph. A node in the cluster graph is also called a cluster, and thus we used the term cluster and nodes interchangeably.

We apply Maximum a Posteriori (MAP) inference to find values of random variables, i.e., subunit conformations that maximize the score (Eq. 5). The MAP inference is applied using max-sum belief propagation (Koller and Friedman, 2009), which is a message-passing algorithm. To apply max-sum belief propagation, we first generate a cluster graph from the MRF graph to transfer information between nodes. A cluster graph has subunit/single clusters (nodes) representing poses of subunits relative to the EM map and pairwise/interaction nodes representing interactions between subunits. There is an edge between single and pairwise nodes if the pairwise node includes the subunit (the diagram on the right in Figure 1). The max-sum belief propagation method works by assigning initial beliefs to all nodes and then sending messages in two phases (Figure 2). The initial beliefs for single nodes and pairwise nodes are the weighted sums of the goodness-of-fit scores of candidate poses for single nodes and the weighted sums of interaction scores between candidate poses of pairs of single nodes for pairwise nodes. After computing the beliefs of candidate poses, each single node sends its beliefs to all connected pairwise nodes. Subsequently, pairwise nodes send messages back to their neighboring single nodes. A message from a pairwise node (*i*, *j*) to a single node *i* is the maximum of the sum of the belief of the pairwise interaction and the message received from single nodes. Thus, for each pose of subunit *i*, the pairwise node (*i*, *j*) sends its pairwise belief with the pose of subunit *i* and the belief of subunit j’s pose that maximizes the sum of the beliefs. Single nodes add received messages to their beliefs. From the final beliefs of single nodes, we can extract the top-scoring pose. We check if subunits do not have a significant amount of atom clashes overall in the complex.

Our goal is to find the top K docking conformations (in this work K = 10). To extract the top 10 conformations efficiently, we used a max-heap tree, an efficient algorithm for finding maximum values (Cormen et al., 2009) (Supplementary Figure S1).

Representing the fitting problem using MRF reduces the computational time. The brute force method of fitting subunits into a density map would be to enumerate all possible combinations of poses of all individual subunits. The advantage of MRF is that information/scores of the various conformations are exchanged in two phases: from single nodes to pairwise nodes, and then from pairwise nodes to single nodes. Thus, instead of an exhaustive search, docking conformations are considered for every pair of subunits to decide the most plausible pose of the individual subunits. After subunits receive all the information about the other subunits and interactions, extracting top conformations is performed using an efficient max-heap tree, which has a constant time for selecting the top-scoring conformation and subsequent conformations in a log time.

### 2.2 Evaluation Metrics

We evaluated the resultant final docked conformations in terms of the root-mean-square deviation (RMSD) from the native structure and the Assembly Placement Score (APS). The RMSD is computed between the $C_{\alpha }$ atoms of the modeled structure and the reference structure. If a structure has identical subunits, the minimum RMSD among permutations is considered. The APS measures the average distance and angle deviation needed to superimpose each subunit of the resultant model into the reference structure, weighted by the number of residues of each subunit.

## 3 Results

### 3.1 Structure Fitting Results

We benchmarked the performance of MarkovFit on three datasets: a set of 31 simulated EM maps; a set of nine high-resolution experimentally determined cryo-EM maps; and a set of 28 experimentally determined cryo-EM maps of medium to low resolution. All datasets are non-redundant, e.g., any protein pairs from two maps have less than 25% global sequence identity between each other.

The simulated EM maps of 31 protein complexes were constructed as follows: From the Protein Data Bank (PDB) (Berman et al., 2000), we selected entries that contain only proteins and have two to seven subunits, which yielded 295 entries. Then, we applied the 25% sequence identity cutoff, which resulted in 22 entries. Then, we added 9 complexes that were common in datasets used in the two previous works (Pandurangan et al., 2015; Bonomi et al., 2019), which we later compared against. EM maps of these complexes were simulated using the epdb2mrc.py program of the EMAN2 package (Tang et al., 2007) at 10 Å resolution and with a voxel size of 1.0 Å.

The high-resolution experimental dataset includes nine cryo-EM maps from EMDB and was selected as follows: We selected EM maps that have associated PDB entries that have three to five protein subunits and resolution less than 4 Å. Next, we applied the 25% sequence identity cutoff. After that, we checked experimental cryo-EM maps and their associated PDB entries manually using UCSF Chimera (Pettersen et al., 2004) to ensure they had sufficient overlap. The contour threshold used for this dataset was 0.5 * the recommended contour level.

The medium to low resolution experimental dataset contains 28 cryo-EM maps from EMDB and was selected as follows: we selected EM maps that have associated PDB entries containing two to seven protein subunits and resolution ranges between 6 and 20 Å, which returned 58 EM maps. Next, we applied the 25% sequence identity cutoff, which resulted in 36 entries. Last, experimental cryo-EM maps and their associated PDB entries are checked manually by UCSF Chimera to assure they have adequate overlap. The contour threshold used was 0.5 times the recommended contour level.

In addition to the simulated and experimental datasets, we have three more datasets derived from them. In the new datasets, protein subunits are randomly rotated and shifted from their original positions. The purpose of the random transformations of subunits is to objectively test the search method. Without random transformation, the resulting accuracy was substantially high (Supplementary Figure S2 shows the fitting accuracy of the simulated and medium resolution experimental datasets).

#### 3.1.1 Structure Fitting Results for Simulated Maps

Structure fitting results for the 31 simulated map datasets are summarized in Table 1. To remove potential bias to the docked poses of subunits in the PDB files, subunits were randomly rotated and shifted before the fitting process by MarkovFit was applied. In Table 1, two results are shown for each target. On the left are the results of the lowest root-mean square deviation (RMSD) model among the top 10 scored models. On the right, we showed the top-scored model. The top-scored model is the model that had the best score among the models generated. This model does not always be the best in quality in terms of RMSD among all the models generated because often the score could not select the best quality model among the pool. The best model among the top 10-scored models is the best RMSD model among the 10 models selected by the score. Since there are 10 choices to choose from, there is a higher chance that the RMSD value of the best model among the top 10-scored models is better than the top-scored model.

**TABLE 1:** Modeling accuracy in the simulated dataset.

PDB ID	No. subunits	Best model by RMSD among top 10			Top-scored model	
		Rank	RMSD ( Å )	APS ( Å , ° )	RMSD ( Å )	APS ( Å , ° )
1CS4	3	1	1.44	(0.1, 6.48)	1.44	(0.1, 6.48)
1VCB	3	1	1.63	(0.25, 6.6)	1.63	(0.25, 6.6)
2DQJ	3	1	1.01	(0.14, 6.7)	1.01	(0.14, 6.7)
1GPQ	4	1	1.27	(0.16, 5.29)	1.27	(0.15, 5.34)
2BBK	4	4	1.23	(0.17, 5.76)	1.41	(0.19, 5.08)
2BO9	4	1	1.71	(0.05, 7.01)	1.71	(0.05, 7.01)
2GC7	4	1	1.08	(0.04, 3.32)	1.08	(0.04, 3.32)
3GPR	4	1	1.65	(0.06, 7.37)	1.65	(0.06, 7.37)
3UIP	4	1	1.36	(0.02, 6.12)	1.36	(0.02, 6.12)
3VH5	4	1	1.49	(0.1, 5.39)	1.49	(0.1, 5.39)
4HUQ	4	4	10.60	(0.18, 24.73)	15.45	(0.33, 50.25)
4WQO	4	1	1.52	(0.44, 5.85)	1.52	(0.44, 5.85)
4WY4	4	3	36.29	(0.84, 104.61)	37.50	(5.34, 91.82)
5FN5	4	6	9.41	(0.06, 16.72)	12.06	(1.27, 19.01)
6VK0	4	1	2.02	(0.11, 5.46)	2.02	(0.11, 5.46)
6ZMS	4	2	22.33	(8.0, 54.4)	22.63	(7.03, 59.17)
7BTY	4	1	1.69	(0.12, 6.55)	1.69	(0.12, 6.55)
7KPX	4	1	2.58	(0.95, 5.39)	2.58	(0.95, 5.39)
4AQ9	5	1	1.91	(0.82, 6.35)	1.91	(0.82, 6.35)
6E14	5	1	2.27	(0.04, 6.08)	2.27	(0.04, 6.08)
6FCZ	5	1	2.17	(0.21, 8.44)	2.17	(0.21, 8.44)
6OWO	5	2	6.25	(0.42, 14.25)	6.48	(0.25, 18.59)
6RD6	5	6	23.33	(4.28, 29.93)	29.28	(2.11, 50.58)
6SGX	5	1	2.17	(0.11, 5.13)	2.17	(0.11, 5.13)
1MDA	6	2	1.43	(0.12, 5.31)	5.85	(0.01, 15.65)
6ND1	6	1	5.00	(0.09, 11.51)	5.00	(0.09, 11.51)
6O22	6	1	7.38	(0.46, 19.99)	7.38	(0.46, 19.99)
7JTI	6	1	2.23	(0.08, 6.78)	2.23	(0.08, 6.78)
1K8K	7	6	5.61	(0.43, 6.07)	11.69	(0.14, 19.99)
1TYQ	7	1	1.99	(0.38, 5.57)	1.99	(0.38, 5.57)
6EHR	7	1	1.70	(0.23, 6.44)	1.70	(0.23, 6.44)

Before structure fitting, individual subunits were randomly rotated and shifted. RMSD for a model was computed relative to the entire complex of the PDB entry after superimposition. No. subunits, the number of subunits in the complex. APS, Average Placement Score, shows the average deviation of subunits in terms of the shifted distance of the subunit center and the rotation angle. Deviation and the angle values for a complex are weighted by the number of amino acids of subunits.

Overall, fitting was very successful in this simulated map dataset. When the best model among the top 10-scored models was considered, 22 out of 31 models (71.0%) had an RMSD of less than 3.0 Å with an average RMSD of 1.69 Å. We also reported the assembly placement score (APS) (Lasker et al., 2009). APS for a complex model is the average distance and angle deviation of subunits in the complex from the reference structure, weighted by the number of amino acids of each subunit. Since APS is weighted by the subunit size, misplacement of a small subunit does not affect the score as much as that of a large subunit. The average APS for the 22 models with a less than 3.0 Å RMSD was 0.21 Å and 6.06 ° for the distance and the angle deviation, respectively. There are five other targets which had an RMSD of less than 10 Å. The average distance and angle deviation of this group were 0.29 and 13.71, respectively. Thus, for all these cases that had an RMSD less than 10 Å, subunits were placed almost at the right place in the density map but often with a relatively deviated angle, which elevated the overall RMSD.

Turning attention now to the top-scored model results (the right column in Table 1), RMSDs were almost identical to the results shown for the best model among the top 10-scored models. Thus, the scoring function was very successful in selecting the best model. Out of the 27 targets that have a less than 10 Å RMSD model within the top 10-scored models, there were only two targets, 5FN5 and 1MDA, where the RMSD of the top-scored model dropped more than 2.0 Å from the best among the top 10.

**FIGURE 3:**
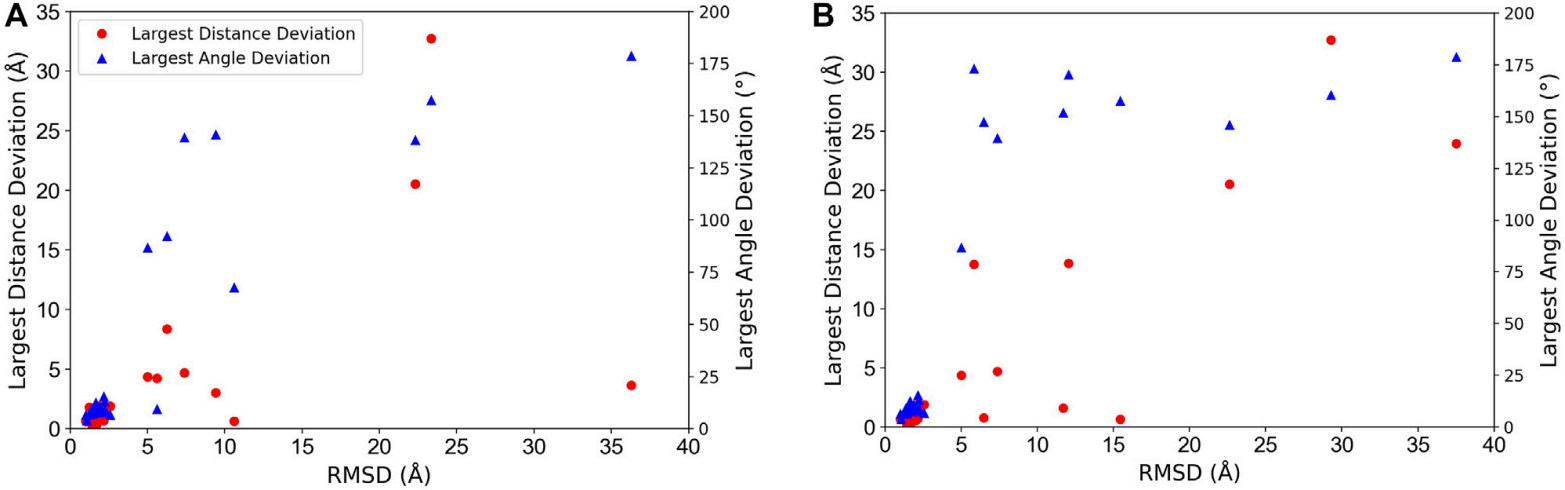
Relationship between overalls RMSD of protein complex models and distance and angle deviation of subunits. The worst (largest) deviations among subunits were plotted. Red circles, distance deviation; blue triangles, angle deviation. **(A)** The best model among the top 10-scored models was considered. **(B)** The top-scored models were considered.

In Figure 3, we analyzed how the placement of individual subunits affects the overall accuracy of the complex model. For each target, the largest distance and angle deviations among subunits were plotted relative to the overall RMSD of the model. Obviously, both distance and angle deviations were small when the overall RMSD was small enough, for example, less than 3 Å, while above 3 Å RMSD, the angle deviation sharply increased above 75°. Also, the distance deviation and RMSD.

**FIGURE 4:**
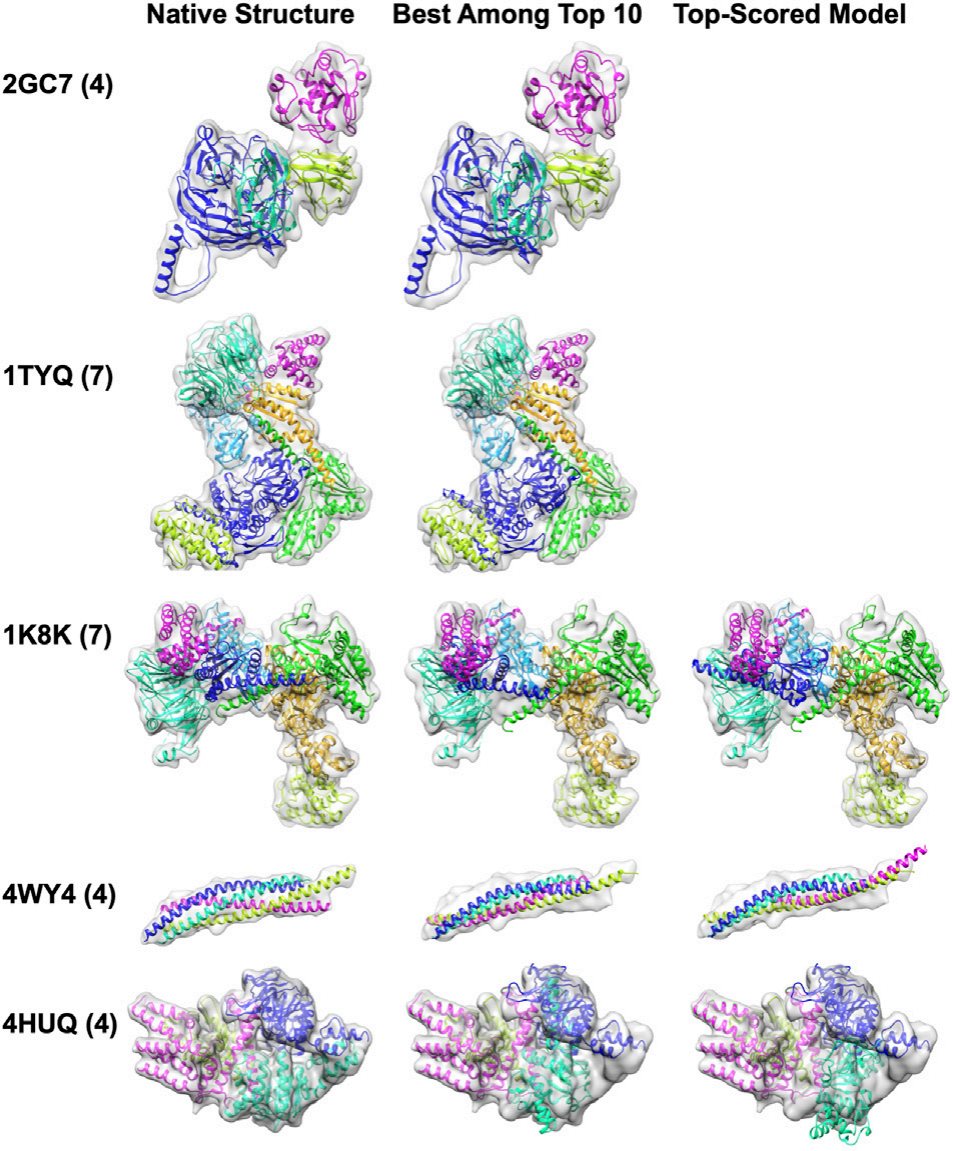
Structure fitting examples of simulated map targets. The number of chains in a complex is shown in the parentheses. The middle panel shows the best among the top 10-scored models, and the right panel shows the top-scored models. Only one fitted structure was shown for 2GC7 and 1TYQ because the top-scored model was the best among the top 10-scored models. 2GCN, the human RhoC-GDP complex. 1TYQ, Arp2/3 complex with bound ATP or ADP. 1K8K, bovine Arp2/3 complex. 4WY4, an autophagic SNARE complex. 4HUQ, folate ECF transporter from *Lactobacillus brevis*.

We show five examples of structure fitting by MarkovFit in Figure 4. The first two targets were successful examples of MarkovFit. For these two targets, 2GC7 and 1TYQ, the best (smallest RMSD) model was ranked at the top by the score, and the RMSD was 1.08 Å and 1.99 Å, respectively. For the third example, a seven-chain complex of actin-related protein (Arp) 2/3 complex (1K8K), there was a 5.61 Å RMSD model ranked within the top 10; however, it was not selected as the top-scored model. The top-scored model had an RMSD of 11.69 Å. For this target, the main difficulty was placing Chain F (dark blue) in the correct position and orientation. In the top-scored model, Chain F was placed around at the right position (1.62 Å) but with a large rotation deviation of 151.81°. The best model among the top 10 models had an APS of (4.23 Å, 9.41 ° ). The next example is the autophagic SNARE complex (4WY4), which has a four-helix bundle structure. This was a difficult target for structure fitting because four helixes are hard to distinguish. In particular, two subunits, Chain C (yellow) and Chain D (magenta), have large rotation deviations of 178.7° and 172.0°, respectively, for the best among the top 10-scored models, resulting in a large RMSD of 36.29 Å. In the top-scored model, Chain D was shifted by 24.0 Å, which further increased the RMSD to 37.5 Å. For the last example, the folate ECF transporter (4HUQ), the main problem was Chain B (cyan). Although this chain was placed almost in the right position (distance deviations of 0.04 Å and 0.49 Å in the best among the top 10-scored model and the top-scored model, respectively), it had a large angle deviation (67.7° and 157.5° in the best among the top 10-scored model and the top-scored model, respectively), which made RMSD over 10 Å.

#### 3.1.2 Structure Fitting Results for Experimental Maps

##### 3.1.2.1 Fitting to High Resolution Maps

Although the main purpose of MarkovFit is to fit individual subunits into medium resolution density maps, we first tested the algorithm on higher resolution maps. For that, we generated a dataset of nine experimental cryo-EM maps with a resolution of less than 4 Å.

The results are summarized in Table 2. Table 2 shows two results for each target. On the left, we showed the results of the lowest RMSD model among the top 10 scored models. On the right, we showed the results of the top-scored model. Fitting was highly successful in this dataset. Looking at the lowest RMSD model among the top 10-scored models, all nine models had an RMSD of less than 3.0 Å with an average RMSD of 1.86 Å and average APS of 1.11 Å and 5.32 ° for the distance and the angle deviation, respectively. The results of the top-scored models are identical to the lowest RMSD model among the top 10 scored models, indicating that the scoring function was able to identify the best model among the top 10 models.

**TABLE 2:** Modeling accuracy in the high-resolution experimental map dataset.

EMDB ID	PDB ID	No. subunits	Res. ( Å )	Voxel size ( Å )	Best model by RMSD among top 10			Top-scored model	
					Rank	RMSD ( Å )	APS ( Å , ° )	RMSD ( Å )	APS ( Å , ° )
9108	6MEO	3	3.9	1.059	1	1.94	(0.42, 5.88)	1.94	(0.42, 5.88)
13508	7PM0	3	3.6	1.1	1	1.64	(0.18, 7.15)	1.64	(0.18, 7.15)
25368	7SP8	3	2.7	1.08	1	1.29	(0.32, 4.61)	1.29	(0.32, 4.61)
30093	6M5U	3	3.8	1.062	1	2.36	(0.17, 4.84)	2.36	(0.17, 4.84)
21897	6WTI	4	2.38	1.08	1	1.31	(7.78, 4.01)	1.31	(7.78, 4.01)
23827	7MGE	4	3.94	0.94	1	1.87	(0.17, 6.2)	1.87	(0.17, 6.2)
30614	7D8X	4	2.6	1.0825	1	1.96	(0.35, 4.86)	1.96	(0.35, 4.86)
22417	7JPO	5	3.2	1.07	1	2.54	(0.36, 6.17)	2.54	(0.36, 6.17)
25426	7STE	5	2.73	0.826	1	1.74	(0.21, 4.17)	1.74	(0.21, 4.17)

No. subunits, the number of subunits in the structure; Res., reported resolution of the maps; voxel size, the size of the grid voxels of the maps. As we showed in Table 1, two results are shown: the best RMSD, model among the top 10-scored models and the top-scored models.

##### 3.1.2.2 Fitting to Medium Resolution Maps

Next, we tested MarkovFit on the 28 experimental map datasets (Table 3). Here, again, we randomly rotated and shifted subunits before applying the structure fitting process to avoid bias in the initial poses of subunits in the PDB files. Similar to the simulated map cases, the resulting accuracy was substantially higher if random transformation was not applied (Supplementary Figure S3).

**TABLE 3:** Modeling accuracy in the medium resolution experimental map dataset.

EMDB ID	PDB ID	No. subunits	Res. ( Å )	Voxel size ( Å )	Best model by RMSD among top 10			Top-scored model	
					Rank	RMSD ( Å )	APS ( Å , ° )	RMSD ( Å )	APS ( Å , ° )
3658	5NL2	2	6.6	1.35	1	2.44	(0.34, 5.51)	2.44	(0.34, 5.51)
22647	7K2V	2	6.6	1.05	2	24.71	(19.9, 153.6)	25.29	(22.3, 160.53)
30324	7CA5	2	7.6	1.06	1	3.29	(0.99, 5.39)	3.29	(0.99, 5.39)
8673	5VH9	2	7.7	1.2	1	0.96	(0.24, 2.29)	0.96	(0.24, 2.29)
8898	6AR6	2	9	3	1	2.20	(0.79, 6.75)	2.20	(0.79, 6.75)
7327	6C13	2	11.33	1.72	2	2.24	(5.09, 12.82)	2.29	(2.09, 12.85)
5450	3J1Z	2	13	2.735	2	21.73	(5.73, 82.88)	32.33	(1.45, 85.73)
0366	6N88	3	6.2	1.43	9	6.55	(0.05, 10.61)	7.33	(0.13, 13.78)
3445	5M5N	3	9.3	2.2	2	18.60	(1.07, 64.62)	34.63	(3.87, 63.73)
1495	3CRF	3	17	1.591	3	3.09	(0.76, 23.91)	30.15	(41.1, 106.63)
3329	5FVM	4	6.1	1.33	1	2.77	(0.13, 5.84)	2.77	(0.13, 5.84)
6476	3JBR	4	6.1	1.32	2	14.90	(1.65, 18.55)	45.60	(7.99, 97.8)
2526	4CHV	4	7	0.975	4	2.32	(0.31, 6.94)	2.81	(0.14, 6.94)
8097	5IOU	4	7	1.07	1	3.67	(0.48, 8.49)	3.67	(0.48, 8.49)
3340	5FWP	4	7.2	1.315	10	13.19	(0.27, 18.03)	16.54	(2.43, 19.83)
8475	5U05	4	7.9	1.26	9	2.32	(0.1, 6.39)	2.55	(0.07, 6.39)
21617	6WCQ	4	8.5	2.28	5	17.24	(3.86, 17.47)	34.54	(15.22, 38.45)
8091	5IDF	4	10.31	1.76	1	4.36	(0.38, 6.58)	4.36	(0.38, 6.58)
6553	3JCH	5	7.06	1.352	3	2.84	(0.15, 5.16)	6.68	(0.65, 7.21)
3201	5FKU	5	8.34	1.76	1	1.78	(0.04, 4.79)	1.78	(0.04, 4.79)
10255	6SN9	5	9.8	1.065	5	1.94	(1.84, 5.22)	7.55	(2.05, 11.54)
2355	4BIJ	5	16	4.4	10	17.09	(4.58, 43.52)	36.61	(3.04, 88.15)
8796	5WCB	6	6	1.31	2	1.94	(0.2, 5.79)	2.09	(0.03, 6.04)
7066	6B7Z	6	6.5	1.073	3	2.47	(0.12, 6.61)	2.64	(0.04, 6.62)
3435	5G4G	6	7.8	1.45	1	2.35	(0.2, 5.59)	2.35	(0.2, 5.59)
3087	5A9K	6	19	1.6	8	24.76	(3.17, 104.07)	27.63	(15.7, 103.71)
1940	3ZW6	6	20	3.308	10	22.95	(3.96, 74.36)	25.36	(5.57, 98.17)
6671	5XF8	7	7.1	1.32	1	3.22	(0.16, 7.39)	3.22	(0.16, 7.39)

No. subunits, the number of subunits in the structure; Res., reported resolution of the maps; voxel size, the size of the grid voxels of the maps. Two results are shown: the best RMSD, model among the top 10-scored models and the top-scored models.

Clearly, structure fitting was more difficult on this dataset as compared with the previous simulated map dataset and the high-resolution experimental dataset: when the lowest RMSD model among the top 10-scored models was considered, 13 out of 28 models (46.4%) had an RMSD of less than 3.0 Å with an average RMSD of 2.20 Å. When counted on the simulated map dataset (Table 1), the fraction was 71.0%. There were six more targets with an RMSD of less than 10 Å, which had an average RMSD of 4.03 Å. We tried to refine the model structures with the Rosetta Relax protocol (Nivon et al., 2013) and a rigid-body refinement (Esquivel-Rodriguez et al., 2012) but did not observe meaningful improvement (data not shown). The conformational change that the refinement methods could make was not large. Thus, if a model’s RMSD is large, it is often beyond what the refinement methods can handle. Also, changes made by the refinement methods were not always in the right direction and made the structure worse.

The scoring function worked well on this dataset as well. Looking at the top-scored model results, RMSD values were identical or close to the best model among the top 10-scored models, especially for targets where the best model among the top 10 has an RMSD less than 10 Å. Out of the 19 targets that have a less than 10 Å RMSD model within the top 10-scores, the score was able to select the lowest RMSD model as the top for 16 of them (84.2%).

**FIGURE 5:**
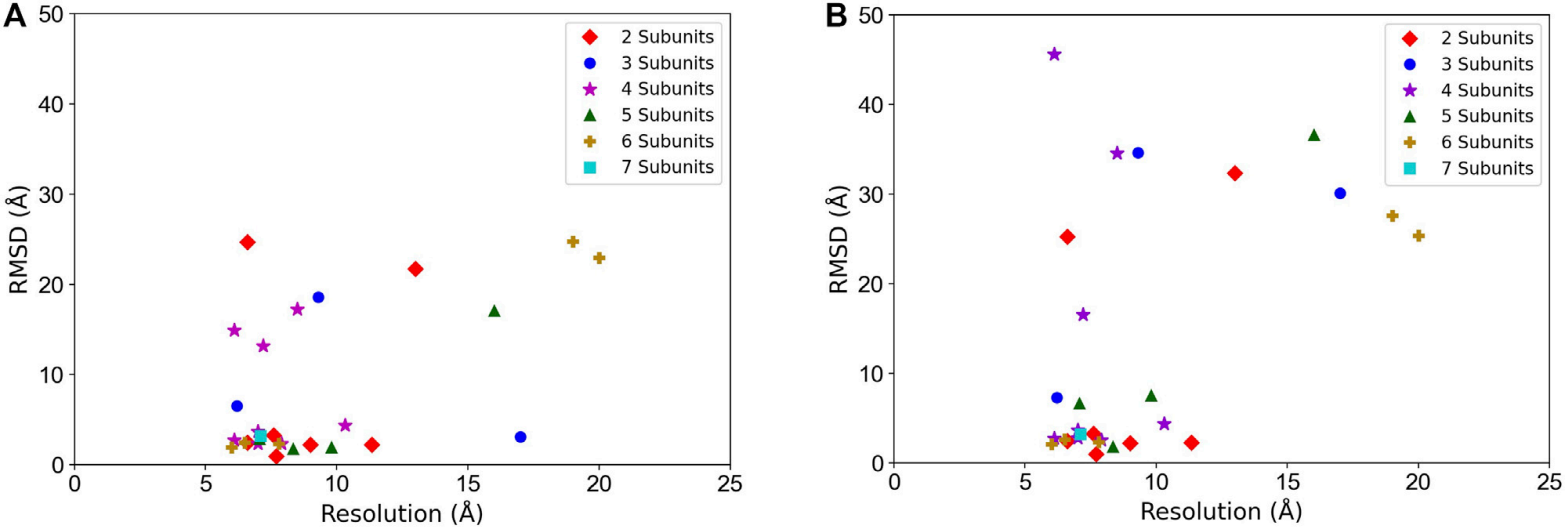
Structure fitting accuracy relative to the map resolution. RMSD of the entire model structure of complexes is plotted. **(A)** the best RMSD model among the top 10-scored models. **(B)** the top-scored models. Targets with different chain numbers are separately plotted.

In Figure 5, we examined the correlation between the map resolution and the model accuracy. There is no clear linear correlation between the model RMSD and the map resolution observed. But we can see that the structure fitting accuracy substantially dropped when the map resolution was worse than 10 Å. For example, considering the best among the top 10 models, when the map resolution is better than 10 Å, 16 out of 21 maps (76.2%) had a model with an RMSD lower than 5 Å but it drops to 42.9% (3 out of 7 maps) when maps with a resolution worse than 10 Å are counted.

**FIGURE 6:**
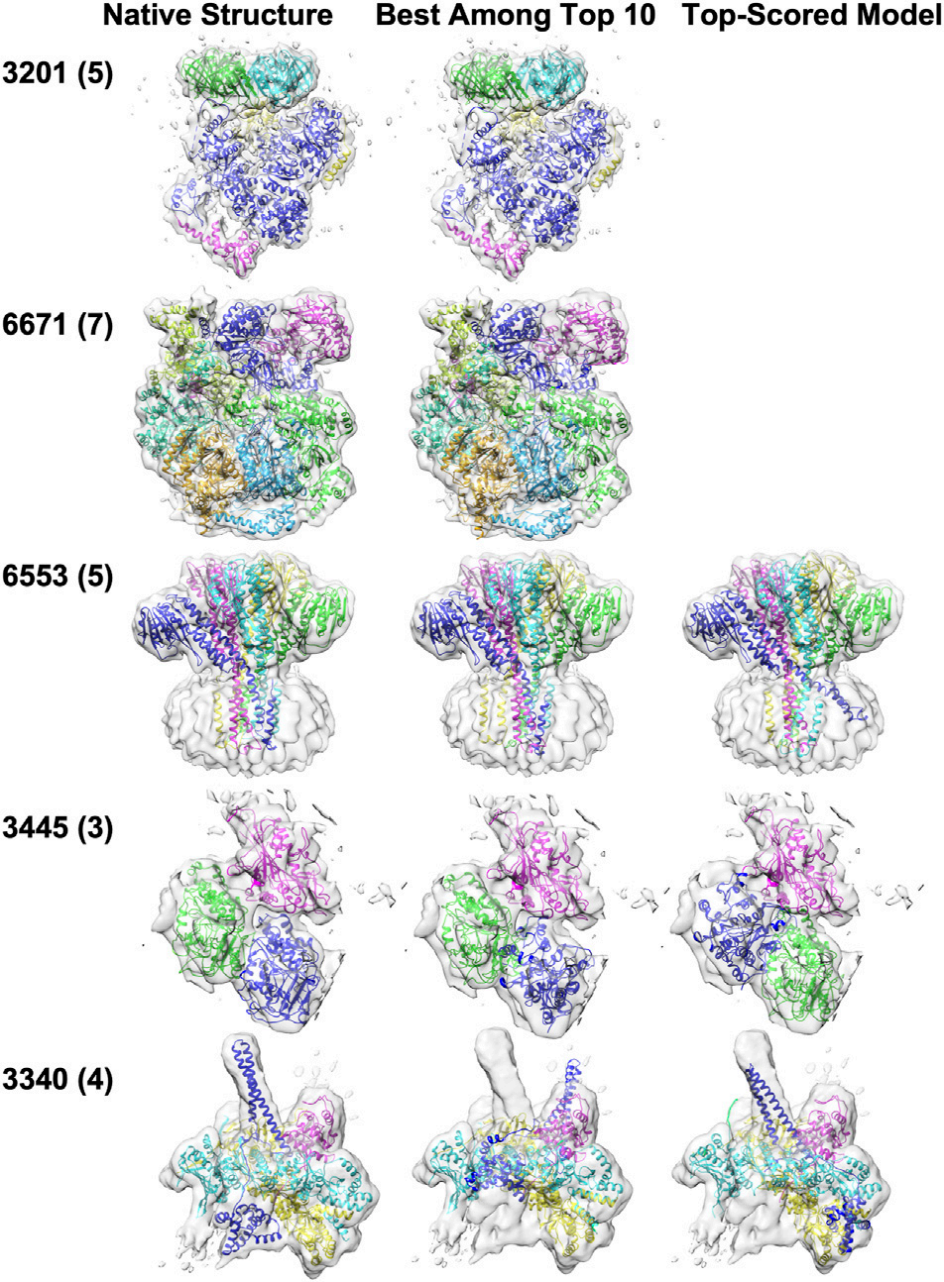
Structure fitting examples of experimental map targets. The number of chains in a complex is shown in the parentheses. The middle panel shows the best among the top 10-scored models, and the right panel shows the top-scored models. Only one fitted structure was shown for EMD-3201 and EMD-6671 because the top scored model was the best among top 10-scored models. (EMD-3201, PDB ID: 5FKU), the *E. coli* replicative DNA polymerase complex in DNA free state. (EMD-6671, PDB ID: 5XF8), the Cdt1-MCM2-7 complex in the AMPPNP state. (EMD-6553, PDB ID: 3JCH), the magnesium channel CorA in the magnesium-free asymmetric open state II. (EMD-3445, PDB ID: 5M5N), microtubule-bound *S. pombe* kinesin-5 motor domain in the AMPPNP state. (EMD: 3340, PDB ID: 5FWP), the Hsp90-Cdc37-Cdk4 kinase complex.

Five examples of structure fitting to experimental maps are presented in Figure 6. MarkovFit worked well in the first two targets, the *E. coli* replicative DNA polymerase complex in DNA-free state (EMD-3201, PDB ID: 5FKU) and the Cdt1-MCM2-7 complex in the AMPPNP state (EMD-6671, PDB ID: 5XF8). Near-native complex structure was successfully ranked as the top score, which has an RMSD of 1.78 Å and 3.22 Å, respectively. For the third example, a homo five-chain complex of CorA from *Thermotoga maritima* in the absence of magnesium, state II (EMD-6553, PDB ID: 3JCH), the best-RMSD model among the top 10 models has an RMSD of 2.84 Å, but it was not selected as the top-scored model. The top-scored model had an RMSD of 6.68 Å. This is a membrane protein complex, and the large volume of the density in the bottom half of the map is mostly from the nanodiscs, which mimic the membrane environment. Among the top 10 models, the best model ranked third by the score placed all subunits in their correct position and orientation. However, the top-scored model had one chain (blue) in a substantially deviated orientation, which caused a larger overall RMSD of 6.68 Å. The next example is the microtubule-bound *S. pombe* kinesin-5 motor domain in the AMPPNP state (EMD-3445, PDB ID: 5M5N), a three-chain complex. In the best among the top 10-scored model which has an RMSD of 18.60 Å, Chain A (blue) was placed around the right position but with a large rotation deviation of 166.22°. In the top-scored models, Chain A (blue) was placed at the position of Chain B (a shift of 29.76 Å) with a large rotation deviation of 178.84°. In turn, Chain B (green) was placed in the position of Chain A (a shift of 18.68 Å) with a small rotation deviation of 2.83°, resulting in the increase in the RMSD value of the top-scored model to 34.63 Å. The last example is the Hsp90-Cdc37-Cdk4 kinase complex (EMD: 3340, PDB ID: 5FWP), which has four chains. The main difficulty with this target was Chain E (blue). For this target, both the best among the top 10-scored models and the top-scored model have an RMSD above 10 Å. In the best among the top 10-scored models, Chain E (blue) did not occupy its density well due to a large rotation of 50.45°. In the top-scored model, Chain E (blue) was shifted by 14.62 Å and has a rotation deviation of 65.91°.

### 3.2 Comparison With Other Methods

We compared MarkovFit with two state-of-the-art methods for structure fitting. The first method is γ -TEMPy, which works by first identifying feature points representing atomic unit centroids in a density map using vector quantization and then applying a genetic algorithm to build structural models (Pandurangan et al., 2015). The second method, designed by Bonomi et al., uses a Gaussian mixture model to represent a density map and a Bayesian scoring function to rank the generated models (Bonomi et al., 2019).

The comparison was performed on a dataset of simulated maps at 10 Å as shown in Table 4. This dataset includes nine protein complexes, which are in common with Table 1 and the datasets used in the two works (Pandurangan et al., 2015) and (Bonomi et al., 2019). In addition, from the γ -TEMPy dataset (Table 1 in their work), we added the remaining one protein complex and the remaining 10 protein complexes from Bonomi’s dataset (Table 2 in their work). As we presented in Tables 1, 2, two data points are shown for each target; the best RMSD among the top 10-scored models and the RMSD of the top-scored model.

**TABLE 4:** Comparison with the two existing methods on simulated maps at 10 Å resolution.

PDB ID	No. subunits	Best RMSD (Å) of the among top 10-scored model			RMSD (Å) of the top-scored model		
		MarkovFit	γ -TEMPy	Bonomi	MarkovFit	γ -TEMPy	Bonomi
1CS4	3	1.43	4.0	(2.6) (159)	1.43	4.0	3.5
1GPQ	4	1.26	3.2	(1.7) (805)	1.26	3.3	2.2
1MDA	6	1.43	14.1	(7.8) (4286)	5.85	14.1	8.1
1TYQ	7	1.99	16.9	(19.1) (599)	1.99	34.8	19.8
1VCB	3	1.62	(7.7) (16)	(1.8) (453)	1.62	25.3	2.2
2BBK	4	1.22	10.9	(2.1) (857)	1.41	14.9	2.4
2BO9	4	1.70	3.3	(1.3) (502)	1.70	9.9	1.6
2DQJ	3	1.00	3.5	(2.0) (89)	1.00	3.9	2.5
2GC7	4	1.07	11.9	(1.3) (817)	1.07	22.5	2.0
1SGF	6	13.05	16.7	-	15.38	20.5	-
1SUV	6	1.79	-	(5.2) (247)	1.79	-	5.3
1Z5S	4	2.18	-	(8.7) (892)	2.18	-	9.0
2UZX	2	2.10	-	(1.1) (551)	2.10	-	1.5
2WVY	3	2.32	-	(0.9) (480)	4.56	-	1.3
3LU0	5	2.93	-	(9.0) (800)	9.70	-	9.3
3NVQ	4	2.10	-	(0.9) (903)	2.10	-	1.0
3PDU	4	2.36	-	(1.3) (802)	1.81	-	1.6
3PUV	5	2.38	-	(22.7) (698)	2.38	-	23.4
3R5D	3	1.52	-	(1.4) (63)	1.52	-	1.9
3SFD	4	19.59	-	(1.4) (385)	21.91	-	1.5

The results of γ -TEMPy and Bonomi’s method are taken from their articles. This dataset was constructed by combining the datasets used in these two articles (Table 1 in the **γ**-TEMPγ article (*Pandurangan et al., 2015*) and Table 2 in the Bonomi’s method (*Bonomi et al., 2019*). “-” in the table indicates that the target was not included in the paper. In the left column, if the score rank of the reported model is above 10, we use (RMSD) (rank) to show the RMSD and the rank of the best-RMSD model.

When the top-scored models of the nine common targets were considered, MarkovFit clearly showed the best performance. The average RMSD of these targets is 1.93 Å, 14.74 Å, and 4.92 Å for MarkovFit, γ-TEMPy, and the Bonomi’s method, respectively. MarkovFit showed the lowest RMSD for eight out of the nine targets. Our method had a substantially higher accuracy on 1TYQ, where our method had an RMSD of 1.99 Å and the other two methods had over 10 Å RMSD.

We also compare MarkovFit with each of the two methods individually in terms of the top-scored models. 1SGF is the remaining target to compare with γ-TEMPy. For this target, both MarkovFit and γ-TEMPy did not produce near-native conformation, but the former had a lower RMSD of 15.38 Å. With the Bonomi’s method, we compare the 19 targets for which both methods have results. The average RMSD values on these targets by MarkovFit and Bonomi’s method were 3.55 Å and 5.27 Å, respectively. MarkovFit had a lower RMSD than the Bonomi’s method for 12 out of the 19 targets.

In terms of the best among the top 10-scored models, MarkovFit and γ-TEMPy had an average RMSD of 2.58 Å and 9.2 Å, respectively, on the 10 targets that are common for these 2 methods. Comparing against the Bonomi’s method is not possible because the reported results in their papers are models ranked lower than the top 10.

### 3.3 Fitting Predicted Models by AlphaFold2

To further investigate the performance of MarkovFit, we tested the method on protein structure models generated by AlphaFold2 (Jumper et al., 2021). AlphaFold2 is a deep learning-based method for predicting protein structure models from sequences that showed a substantial improvement in structure prediction accuracy in the Critical Assessment of techniques in protein Structure Prediction (CASP) (Kryshtafovych et al., 2021).

We modeled individual protein subunits in the medium-resolution experimental dataset by AlphaFold2. Among five models produced by AlphaFold2, we reported the best RMSD in Supplementary Table S1. Among the 28 targets in the dataset, interestingly, there were only six targets where AlphaFold2 modeled all their subunits within an RMSD of less than 5 Å (Supplementary Table S1). We performed structure fitting only for these six targets. Table 5 summarizes the results.

**TABLE 5:** Modeling accuracy using AlphaFold2 subunit models.

EMDB ID	PDB ID	No. Subunits	Res. ( Å )	Voxel size ( Å )	Best model by RMSD among top 10			Top-scored model	
					Rank	RMSD ( Å )	APS ( Å , ° )	RMSD ( Å )	APS ( Å , ° )
30324	7CA5	2	7.6	1.06	1	4.0	(1.53, 9.73)	4.0	(1.53, 9.73)
8475	5U05	4	7.9	1.26	8	2.79	(0.83, 7.86)	3.1	(0.41, 8.21)
3435	5G4G	6	7.8	1.45	9	4.83	(0.39, 7.52)	5.07	(0.43, 5.51)
8796	5WCB	6	6	1.31	-	-	-	-	-
3087	5A9K	6	19	1.6	10	20.6	(1.89, 111.51)	20.82	(1.95, 112.32)
1940	3ZW6	6	20	3.308	2	15.24	(0.57, 169.04)	15.83	(0.62, 167.2)

Among the targets in the medium resolution dataset (Table 3), we only performed fitting for targets that have all subunits modelled by AlphaFold2 within an RMSD, of 5 Å. No. subunits, the number of subunits in the structure; Res., reported resolution of the maps; voxel size, the size of the grid voxels of the maps. Two results are shown: the best RMSD model among the top 10-scored models and the top-scored models. “-” in the table indicates that modeling by MarkovFit did not yield valid models due to clashes.

Among the six targets, only one target had an RMSD of less than 3 Å and three targets were within 10 Å RMSD when the lowest RMSD model among the top 10-scored models was considered. The map EMD-8796 (PDB: 5WCB) did not yield models as subunit poses had too many clashes among them. Thus, overall, AlphaFold2 models were not accurate enough for most of the targets in this dataset.

### 3.4 Computational Time

**FIGURE 7:**
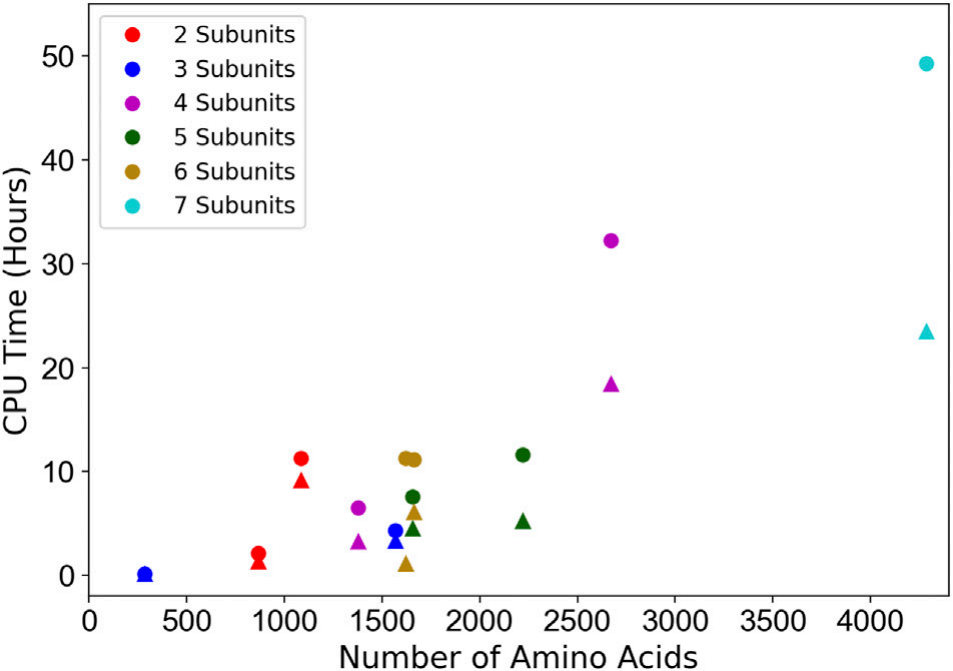
Computational time relative to the protein complex size. The CPU time of the entire process (circles) as well as the initial subunit pose search step (triangles) are shown. 11 experimental map targets with different number of chains were used: EMD-3658, EMD-7327, EMD-0366, EMD-1495, EMD- 2526, EMD-8475, EMD-6553, EMD-3201, EMD-8796, EMD-3087, and EMD-6671. The time was measured on 20 cores of AMD EPYC 7402P 24-Core @ 2.8 GHz.

The CPU hours of running MarkovFit are shown in Figure 7. They were measured on 11 experimental map targets from the medium resolution dataset with a different number of subunits. The computational time of the entire MarkovFit process is the sum of the times for the subunit pose search process using FFT, the pairwise score computation between every pair of subunits, the belief propagation application on the MRF graph, and the step of picking the top 10 docked structures using the max-heap tree. The subunit pose search process and the pairwise score computation take most of the MarkovFit time, while the belief propagation algorithm and the picking of the top 10 conformations step take a few minutes. The search process and the pairwise score computation on the 11-target experimental subset took on average about 59% (7.7 h) and 40% (6.25 h) of the total computational time of MarkovFit, respectively.

In Figure 7, we see a correlation between the number of amino acids in the protein complex and the computational time. The two main factors that affect the computational time are the size of the map and the number of subunits. Apparently, a large map has a larger search space. As the search process is performed on each subunit individually, the running time increases as we have more subunits. Also, the pairwise interaction score computation increases as we have more subunits.

## 4 Discussion

In this work, we developed MarkovFit, a procedure to perform structure fitting to cryo-EM maps of medium to low resolution. The MRF is effective in finding correct combinations of subunit poses that are mapped in the target EM map, and the Max-heap algorithm is used to select top-scored conformations.

Although the algorithm worked well overall, there were several reasons that could lead to failure of the search process. A main cause is the small size of a subunit, e.g., ∼100 or fewer residues as observed in the target 4WY4 in the simulated map dataset (Table 1) and 6N88 and 6SN9 in the experimental map dataset (Table 3). Finding the correct pose of a small domain is difficult, especially when the other subunits are large and have a higher density in the corresponding positions in the EM map. Also, apparently, as the map resolution gets worse, density values across the cryo-EM map become undistinguishable, resulting in incorrect predictions of candidate poses for subunits (Figure 5).

Fitting existing subunit structures have been a major strategy in structure modeling for cryo-EM maps of medium to low resolution. This approach is becoming more important now as predicting structures is becoming increasingly reliable by structure prediction methods of the new generation (Jumper et al., 2021), although AlphaFold2 did not work that well in the dataset we used (Table 5, Supplementary Table S1). Structure models of dozens of organisms are precomputed and made available to the public, too (Aderinwale et al., 2022; Varadi et al., 2022). A future important direction of development would be to combine these two approaches effectively: structure prediction of individual proteins and combining and fitting them into a cryo-EM map.

## Data Availability

The data presented in this study can be found at https://zenodo.org/record/6509947. The MarkovFit code is freely available for academic use *via* https://github.com/kiharalab/MarkovFit.
