# Supplementary material for: MarkovFit: Structure Fitting for Protein Complexes in Electron Microscopy Maps Using Markov Random Field
Source: Front Mol Biosci. 2022 Jul 25;9:935411. doi: 10.3389/fmolb.2022.935411 (PMC9358042; doi:10.3389/fmolb.2022.935411)
Supplement: Supplementary file 1 [file DataSheet1.pdf]

Supplementary Material

The Algorithm of Picking Top K Final Conformations Using a Max-Heap Tree

N is the number of subunits

M is the number of beliefs/poses of subunits

1- Sort beliefs of each subunit in descending order

2- Sum $belief_i$ of all subunits as following:

For i from 1 to M:

$$total\_belief_i = \sum_{j=1}^{N} belief_i(subunit_j)$$

3- Generate a max-heap tree H from the total_belief

4- Pick the complex conformation with the highest belief, check for clashes, and add new complex conformations to H as following:

While number of final_conformations < K:

conformation C = extract root of H (highest-belief complex conformation)

If clashes of conformation C < clash_threshold:

Add complex conformation C to the final conformations

Else:

Discard conformation C

Add N new complex conformations to H as following:

For i from 1 to N:

current_indices = subunits' belief (pose) indices of conformation C

current_indices [($subunit_i$, $belief_j$)] = current_indices [$subunit_i$, $belief_{j+1}$]

new_belief = compute total_belief(current_indices)

Add new_belief to H

Supplementary Figure 1. The process of picking the top K docking conformations of a protein complex using a Max-Heap tree (Cormen et al., 2009) works as follows: 1) Poses of each subunit are sorted in a descending order based on their beliefs. 2) Beliefs of all subunits at each index i are added up (total_belief). 3) The sums of beliefs (total_belief) form a max-heap tree (H) and associated with each sum of beliefs is the list of belief indices. 4) After forming the max-heap tree (H), we extract the root of H and check for clashes between subunits in the retrieved structure. If the clashes in the picked conformation are below the clash threshold, we accept the

conformation; otherwise, the conformation is discarded. In both cases, we add to the max-heap tree (H) a set of new conformations equal to the number of subunits. These new conformations are formed by looking at the list of belief indices of the extracted root. We change the index of one subunit at a time in that list to point to the next best belief, compute the sum of beliefs, and add this sum of beliefs to the max-heap tree. The process of extracting the root, checking for clashes, and adding new nodes to the tree is repeated until we have K conformations.

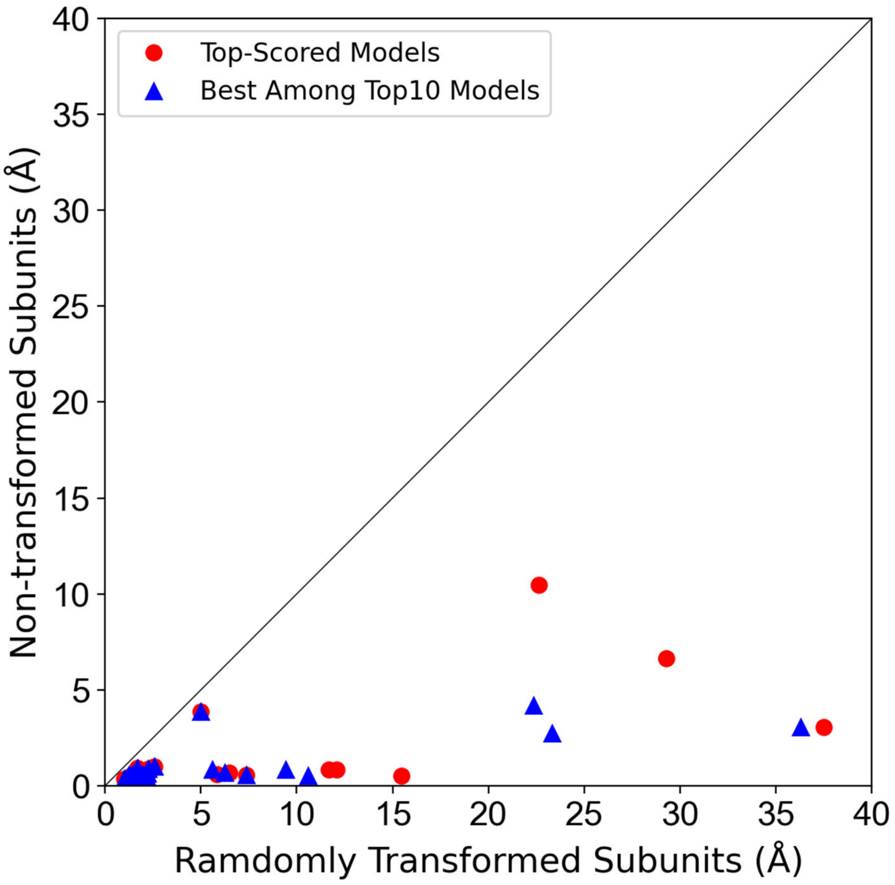

Supplementary Figure 2. Comparison of the fitting accuracy of the simulated dataset in which subunits were randomly transformed versus the simulated dataset of the non-transformed subunits. RMSD of the entire model structure of complexes are plotted. Targets are shown in Table 1.

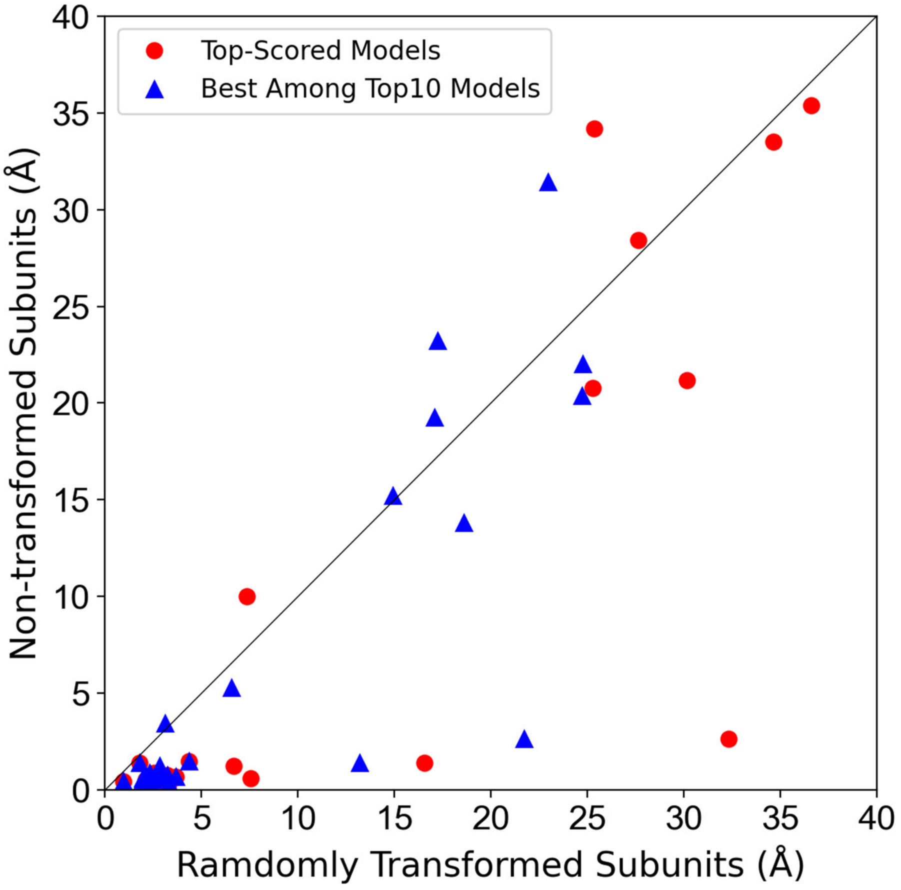

Supplementary Figure 3. Comparison of the fitting accuracy of the medium resolution experimental dataset in which subunits are randomly transformed versus the experimental dataset of the non-transformed subunits. RMSD of the entire model structure of complexes are plotted. Targets are shown in Table 3.

Supplementary Table 1. AlphaFold2 models accuracy of the individual protein subunits in the medium resolution experimental map dataset.

EMDB ID	PDB ID	No. Subunits	Resolution (Å)	Best RMSD (Å) of AlphaFold2 Models
3658	5NL2	2	6.6	12.81 (A, B)
22647	7K2V	2	6.6	4.58 (A)
				22.6 (B)
30324	7CA5	2	7.6	3.96 (A)
				3.04 (B)
8673	5VH9	2	7.7	7.32 (A)
				4.45 (B)
8898	6AR6	2	9	17.86 (A)
				25.12 (B)
7327	6C13	2	11.33	25.41 (A, B)
5450	3J1Z	2	13	5.39 (A, B)
0366	6N88	3	6.2	10.25 (A)
				11.64 (B)
				1.51 (C)
3445	5M5N	3	9.3	2.5 (A)
				1.99 (B)
				5.2 (C)
1495	3CRF	3	17	0.7 (A)
				18.87 (B)
				7.47 (C)
3329	5FVM	4	6.1	-1 (A, B)
				1.87 (C, D)
6476	3JBR	4	6.1	-1 (A)
				2.31 (B)
				5.4 (C)
				-1 (D)
2526	4CHV	4	7	7.25 (A, B, C, D)
8097	5IOU	4	7	5.47 (A, B)
				4.63 (C, D)
3340	5FWP	4	7.2	6.24 (A, B)
				16.99 (C)
				5.45 (D)

8475	5U05	4	7.9	1.42 (A, B, C, D)
21617	6WCQ	4	8.5	2.07 (A)
				1.72 (B)
				8.52 (C)
				1.21 (D)
8091	5IDF	4	10.31	17.63 (A, C)
				14.61 (B, D)
6553	3JCH	5	7.06	5.36 (A, B, C, D, E)
3201	5FKU	5	8.34	9.17 (A)
				1.04 (B, C)
				18.47 (D)
				2.78 (E)
10255	6SN9	5	9.8	9.97 (A)
				3.0 (B)
				12.37 (C)
				2.8 (D)
				6.63 (E)
2355	4BIJ	5	16	19.91 (A, B, C, D, E)
8796	5WCB	6	6	3.15 (A, B, C, D, E, F)
7066	6B7Z	6	6.5	8.09 (A, B)
				2.84 (C, E)
				1.48 (D, F)
3435	5G4G	6	7.8	4.21 (A, B, C, D, E, F)
3087	5A9K	6	19	3.64 (A, B, C, D, E, F)
1940	3ZW6	6	20	3.65 (A, B, C, D, E, F)
6671	5XF8	7	7.1	4.98 (A)
				3.03 (B)
				3.83 (C)
				7.47 (D)
				5.17 (E)
				2.77 (F)
				28.06 (G)

No. Subunits, the number of subunits in the structure; Resolution, reported resolution of the maps. The RMSD of the best-RMSD model among the top five models generated by AlphaFold2 (Jumper et al., 2021) is reported. “()” in the table includes subunit IDs. If the protein complex has identical subunits, these subunits are grouped together on the same row. ”-1” in the table indicates that AlphaFold2 was

not able to provide models for that protein subunit either because the sequence is too long, i.e., it has more than 2400 resides or because it contains unknown residues.

References

Cormen, T.H., Leiserson, C.E., Rivest, R.L., and Stein, C. (2009). *Introduction to algorithms.* Cambridge, Massachusetts: The MIT Press.

Jumper, J., Evans, R., Pritzel, A., Green, T., Figurnov, M., Ronneberger, O., et al. (2021). Highly accurate protein structure prediction with AlphaFold. *Nature* 596(7873)**,** 583-589. doi: 10.1038/s41586-021-03819-2.
